# Prognostic impact of micrometastases and nodal status in early and advanced oral squamous cell carcinoma

**DOI:** 10.1007/s00405-026-10070-w

**Published:** 2026-03-05

**Authors:** Gabriel Johansson, Martin Harper Hysek, Linda Marklund, Rusana Bark, Krzysztof Piersiala

**Affiliations:** 1 Department of Clinical Sciences, Intervention and Technology, Division of ENT Diseases, Karolinska Institutet, Karolinska University Hospital, Huddinge, B61, 141 86 Stockholm, Sweden; 2 Department of Otorhinolaryngology, Karolinska University Hospital, Eugeniavägen 3, 171 76 Solna, Sweden; 3 Department of Pathology and Cytology, Karolinska University Hospital, Eugeniavägen 3, 171 76 Solna, Sweden; 4 Medical Unit for Pathology and Cancer Diagnostics, Karolinska University Hospital, Eugeniavägen 3, 171 76 Solna, Sweden; 5 Medical Unit Head and Neck, Lung and Skin Cancer, Department of Head and Neck Surgery, Karolinska University Hospital, Eugeniavägen 3, 171 76 Solna, Sweden; 6 Department of Surgical Sciences, Section of Otolaryngology and Head and Neck Surgery, Uppsala University Hospital, 751 85 Uppsala, Sweden

**Keywords:** Micrometastasis, Isolated tumour cells, Sentinel lymph node biopsy, Oral squamous cells carcinoma

## Abstract

**Objectives:**

Sentinel lymph node biopsy (SLNB) enhances staging and treatment of oral squamous cell carcinoma (OSCC) by enabling detection of micrometastases (Mi) and isolated tumour cells (ITCs). However, the prognostic significance of these microscopic tumour deposits remains unclear. This study aims to evaluate the impact of Mi, ITCs, and other nodal characteristics on survival and recurrence across all stages of OSCC.

**Methods:**

This retrospective cohort study included all patients with primary OSCC who underwent SLNB at Karolinska University Hospital between March 2019 and October 2024. Clinical and pathological TNM stages were recorded, including the presence of Mi, ITCs, and extranodal extension (ENE). Disease-free survival (DFS) and disease-specific survival (DSS) were analysed using Kaplan–Meier estimates and Cox proportional hazards models.

**Results:**

A total of 259 patients were included. 66.4% presented with early-stage disease (cT1-T2N0). Recurrence occurred in 15.8% of cases. Increasing tumour stage and nodal burden were associated with poorer DFS and DSS (p < 0.0001). Mi was identified in 11.0% of patients with early-stage OSCC and in 3.4% of cases with advanced disease. Three-year DFS was 88.0% in pN0 disease, 100% for pN0itc, 73.2% for pNmi, and 57.9% in patients with macrometastasis (pN +).

**Conclusions:**

Tumour stage, nodal burden and ENE are strong predictors of DFS and DSS in OSCC. Patients with Mi had outcomes comparable to those with pN1 disease, whereas ITCs did not negatively affect prognosis. These findings support classifying Mi as metastatic disease, and underscore the value of SLNB in management across all stages of OSCC.

## Introduction

Oral squamous cell carcinoma (OSCC) is an aggressive form of cancer associated with high morbidity and mortality, and a rising incidence observed in younger, non-smoking patients [1–3]. The presence of lymph node metastasis is a critical factor that has negative prognostic value, guides treatment decisions, and determines follow-up protocols [4–7]. Furthermore, extranodal extension (ENE) is also associated with a higher rate of recurrence and distant metastasis, thus negatively affecting overall survival [8–11].

In the last decade, sentinel lymph node biopsy (SLNB) has become standard of care in early-stage OSCC (T1-T2N0) [12]. In standard histopathological evaluation, only one or two sections of each lymph node are examined. When evaluating a sentinel lymph node biopsy (SLNB), multilevel sectioning is performed, facilitating detection of microscopic tumour deposits [4, 13]. When utilizing single-photon emission computed tomography with computed tomography (SPECT-CT), assessment of individual lymphatic drainage patterns is possible, as well as accurate anatomical location of tumour draining lymph nodes [12, 14]. Studies have shown that even well lateralized, early-stage OSCC can drain to contralateral lymph nodes [14–16]. Moreover, contralateral occult metastasis is relatively common in advanced-stage OSCC (T3-T4 and all N + disease) [12, 17].

A micrometastasis (Mi) has been defined as a metastatic deposit measuring no more than 2 mm in its greatest dimension and exhibiting specific morphological characteristics [18]. Tumour deposits smaller than 0.2 mm are designated isolated tumour cells (ITC) [19, 20]. The clinical significance of Mi and ITCs has not yet been established. In some cancer forms, such as colorectal and breast cancer, Mi appears to negatively impact survival [21, 22]. In OSCC, however, there is no consensus on their prognostic impact. Some studies suggest that the presence of Mi does not affect survival [23–25] whereas others report an association with increased mortality [5, 26, 27]. Several studies have failed to find a significant effect on survival when ITCs are present [5, 6, 24, 26], although it is difficult to draw conclusions, due to small sample sizes.

The inconsistency of these findings may be attributed to differences in histological assessment techniques, sample sizes and inclusion criteria, as well as variation in how ITCs and Mi are classified across studies [4]. Moreover, the current criteria for ITC and Mi were originally defined for breast cancer [18] and may not be entirely applicable to OSCC due to differences in tumour biology.

Considering these contradictory findings, it is necessary to further investigate the clinical implications of ITCs and Mi in OSCC. Understanding their prognostic relevance may refine staging systems and treatment protocols, potentially leading to a more tailored approach to patient management. This study aims to address these knowledge gaps by analysing a comprehensive data set of OSCC cases at Karolinska University Hospital, providing insight into the impact of ITC and Mi on patient outcomes and contributing to the development of standardized guidelines for their assessment in patients with OSCC.

## Methods

### Patient cohort and management protocol for sentinel lymph node biopsy and sentinel lymph node-assisted neck dissection

All consecutive patients with newly diagnosed OSCC and treated with curative intent who underwent SLNB or SLNB-assisted ND at Karolinska University Hospital between March 2019 and October 2024 were eligible for inclusion. Exclusion criteria were non–squamous cell carcinoma (SCC) on final histology of the primary tumour and cases of recurrent disease.

At Karolinska University Hospital, SLNB or SLNB-assisted ND is currently part of our previously described standard protocol for staging and treatment of both early- and advanced-stage OSCC [12, 28]. The standardized pre-operative tumour assessment included fine-needle aspiration cytology (FNAC), tumour biopsy, computed tomography (CT), SPECT-CT and magnetic resonance imaging (MRI) when clinically indicated. SPECT-CT was conducted following submucosal, peritumoral injection of a technetium-99 (Tc-99m)-labelled radiotracer, after a minimum interval of one hour. Surgery was performed within 24 hours after tracer injection.

Resection of the primary tumour was performed as an en-bloc resection with macroscopic margins of at least 10 mm. Our institution adheres to the Swedish national guidelines for head and neck cancer (*Nationellt vårdprogram för huvud- och halscancer*) that recommend SLNB as an alternative staging procedure in OSCC without known nodal involvement (cN0). In addition, SLNB-assisted neck dissection (ND) is recommended for advanced-stage disease (stage III-IV).

### Adjuvant therapy

Adjuvant therapy was administered in accordance with the current Swedish national guidelines. These guidelines recommend postoperative radiotherapy for patients with pN + disease and for stage II tumours with adverse pathological risk factors such as depth of invasion, perineural growth and close surgical margins. Concomitant chemoradiotherapy is recommended in high-risk disease, defined by the presence of ENE and/or positive margins.

### Definition of ITC, micrometastasis, macrometastasis

**Fig. 1:**
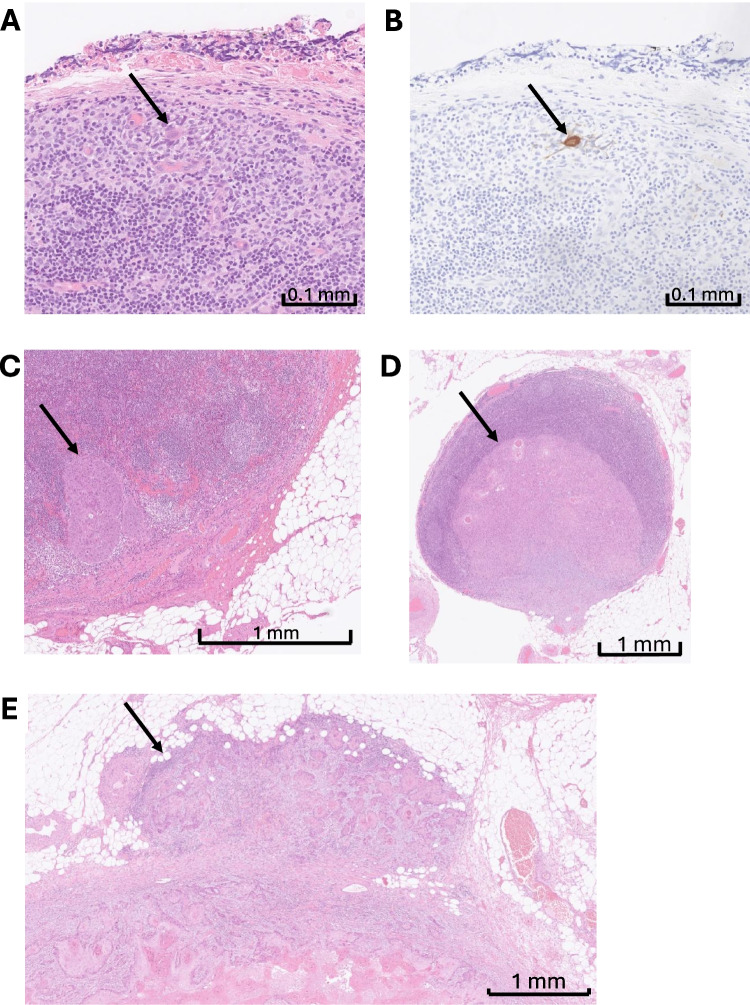
Representative histological examples of each nodal category: Isolated tumour cells (A, B), micrometastasis (C), macrometastasis (D) and extranodal extension (E)

At Karolinska University Hospital, we adhere to established definitions for categorizing nodal metastases in OSCC. In accordance with prior studies and international guidelines, Mi is defined as a metastatic deposit measuring more than 0.2 mm but not exceeding 2 mm in greatest dimension, while ITCs refer to individual or small clusters of tumour cells measuring no more than 0.2 mm. Macrometastasis (also referred to as pN +) denotes nodal metastases larger than 2 mm, and ENE is defined as tumour infiltration extending beyond the lymph node capsule into surrounding tissues. These definitions are consistently applied in our pathological assessments and statistical analyses. Figure 1 illustrates representative histological examples of each nodal category: ITC, micrometastasis, macrometastasis, and ENE. Patients were grouped accordingly in the study as follows: pN0 (no tumour cells), pN0i + (ITCs), pN1mi (Mi), and pN + (macrometastases with or without ENE).

### Statistical methods

All consecutive patients with newly diagnosed primary OSCC – including second primaries – who underwent SLNB or SLNB-assisted neck dissection (ND) at Karolinska University Hospital between March 2019 and October 2024 were included. Cases of recurrent tumours were excluded, as were all non-SCC tumours. The primary endpoints of this study were disease-free survival (DFS) and disease-specific survival (DSS). DFS was defined as the time interval from the date of initial treatment to the date of recurrence of oral squamous cell carcinoma or to the last follow-up. Patients who were lost to follow-up were treated as censored at their last recorded visit. DSS was defined as the time interval from the date of initial treatment to the date of death attributable to oral squamous cell carcinoma. Deaths from other causes and patients who remained alive at the end of the study period were censored at the date of death or last follow-up, respectively.

Survival curves for DFS and DSS were estimated using the Kaplan–Meier method, and differences between groups were assessed using the log-rank test. The ITC group was included in both the Kaplan–Meier survival analyses and log-rank tests to ensure comprehensive evaluation of all histopathological subcategories. Univariate and multivariate Cox proportional hazards regression models were employed to evaluate the prognostic impact of clinicopathological factors, including age (≤ 60 vs. > 60 years), gender (male vs. female), tumour stage (pT1-2 vs. pT3-4), nodal status (pN0 vs. pNmi vs. pN +), ENE status (ENE- vs. ENE +), and overall clinical stage (early vs. advanced). The results were reported as hazard ratios (HRs) with corresponding 95% confidence intervals (CIs). Tumours were staged according to the TNM Classification of Malignant Tumours, 8th Edition, using the unified UICC/AJCC criteria (UICC update October 2020), incorporating depth of invasion (DOI) and extranodal extension (ENE) [20].

pT status and pN status were included in the multivariate Cox regression model to identify independent prognostic factors. To avoid multicollinearity in the multivariate analysis, we included only one significant variable associated with the T stage and one with the N stage, respectively. All statistical analyses were performed using IBM SPSS Statistics for Windows, Version 29.0.1.0 (171) (IBM Corp., Armonk, NY, USA) and GraphPad Prism, Version 10.1.2 (GraphPad Software, San Diego, CA, USA). A two-sided p-value of < 0.05 was considered statistically significant.

### Ethical approval

All procedures involving human participants complied with the ethical standards of the institutional and national research committees and the 1964 Declaration of Helsinki and its later amendments or comparable ethical guidelines. The requirement for written informed consent was waived due to the retrospective design of the study and the absence of any identifiable patient data in the published material. The study was approved by the Swedish Regional Ethical Review Board (approval numbers: 2024–02635-01). Approval date: 2024–09-07.

## Results

### Clinicopathological characteristics and treatment modalities

The study included 259 patients with OSCC (Table 1), with a nearly equal gender distribution (48.3% female and 51.7% male). The majority of patients (66.8%) were over 60 years old. Clinically, 201/259 (77.6%) were classified as cT1-T2, and 205/259 (79.2%) presented with negative nodal status (cN0). Early-stage disease (T1-T2N0) was diagnosed in 172/259 (66.5%), whereas 87/259 (33.6%) had advanced-stage disease (T3-T4 and all N + disease). The most common tumour location was the oral tongue (60.2%), followed by the gingiva (15.1%) and the floor of the mouth (11.2%), with other sites accounting for the remaining cases. There was one case with a tumour on the oral surface of the uvula, which was included as it was p16- and Human papillomavirus (HPV)-negative.

**Table 1:** Demographics and clinical tumour characteristics

Variable	N (%)
**Gender**	
Female	125 (48,3)
Male	134 (51,7)
**Age at surgery**	
< = 60	86 (33,2)
> 60	173 (66,8)
**cT status**	
cT1-T2	201 (77,6)
cT3-T4	58 (22,4)
**cN status**	
cN0	205 (79,2)
cN +	54 (20,8)
**Clinical stage**	
Early stage	172 (66,4)
Advanced stage	87 (33,6)
**Tumour site**	
Tongue	156 (60,2)
Gingiva	39 (15,1)
Floor of the mouth	29 (11,2)
Bucca	24 (9,3)
Retromolar trigone	8 (3,1)
Soft or hard palate	3 (1,2)

Pathological evaluation (Table 2) revealed that 181 (69.9%) tumours were pT1-T2, while 78 (30.1%) were pT3-T4. Nodal involvement was observed in 94 (36.3%) cases, including 67 (25.9%) with macrometastasis (pN +), 22 (8.5%) with only micrometastasis (pNmi), and 5 (1.9%) with isolated tumour cells (pN0itc). 52 out of the total 259 patients (20.1%) were upstaged from cN0 to pN + status, including both macrometastases and Mi. 16 patients (6.2%) were downstaged from cN + (cN1 n = 9; cN2b n = 7) to pN0. Three out of 87 patients (3.4%) with advanced clinical stage disease had nodal Mi, while only one (1.1%) had ITCs. Out of the three patients with Mi, two (2.3%) were clinically staged as N0 and one (1.1%) as N2b, but none of them had macrometastasis upon pathological evaluation. In patients with early-stage disease, 19 out of 172 (11.0%) had Mi, and four (2.3%) had ITCs. ENE was detected in 8.9% of all patients.

**Table 2:** Pathological tumour characteristics. Itc = Isolated tumour cells. Mi = micrometastasis. ENE = Extranodal extension

Variable	N (%)
**pT status**	
pT1	91 (35,1)
pT2	90 (34,7)
pT3	41 (15,8)
pT4	37 (14,3)
**pN status**	
pN0	165 (63,7)
pN0itc	5 (1,9)
pNmi	22 (8,5)
pN1	23 (8,9)
pN2	23 (8,9)
pN3	21 (8,1)
**ENE**	
ENE-	236 (91,1)
ENE +	23 (8,9)
**Recurrence**	
Yes	41 (15,8)
No	218 (84,2)
**Site of recurrence**	
Local	22 (53,7)
Regional	17 (41,5)
Distant	20 (48,8)

The primary assessment modality for the neck was SLNB in 121 (46.8%) cases and SLNB-assisted ND in 138 (53.3%) cases. 128 (49.4%) patients received no adjuvant treatment and were treated with surgery only, while 102 (39.4%) patients received postoperative radiotherapy and 29 (11.2%) received concomitant chemoradiotherapy after primary surgical treatment. The mean follow-up time for surviving patients was 27.6 months (range 3.0–70.0 months), while for deceased patients it was 18.2 months (range 1.4–50.0 months).

### Clinical tumour and nodal staging and survival

**Fig. 2:**
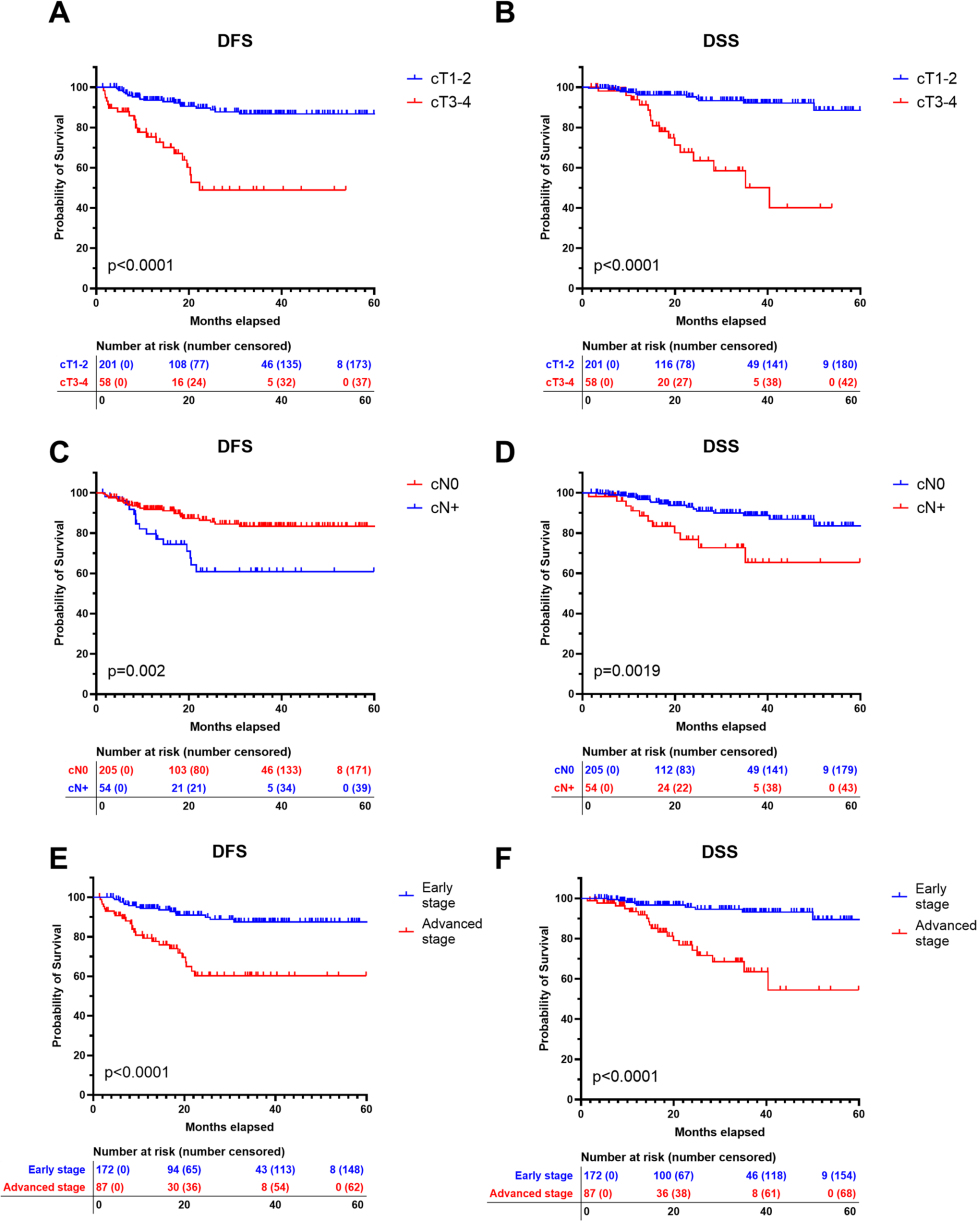
Recurrence and survival in relation to clinical tumour and lymph node characteristics. DFS = Disease free survival. DSS = Disease specific survival

Clinical tumour and nodal staging at the time of diagnosis had a significant impact on survival outcomes (Fig. 2). Patients with smaller tumours (cT1–T2) demonstrated markedly better disease-free survival (DFS) and disease-specific survival (DSS) compared to those with larger tumours (cT3–T4), with 3-year DFS of 86.6% vs 48.9% and DSS of 93.3% vs 50.1%, respectively (Figs. 2A and 2B, p < 0.0001). Similarly, nodal status at presentation was a strong prognostic factor: patients with clinically negative nodes (cN0) had higher 3-year DFS (83.4%) and DSS (88.7%) compared to those with cN + disease (DFS: 60.9%, DSS: 65.4%) (Figs. 2C and 2D, DFS: p = 0.0002; DSS: p = 0.0019). When stratified by overall clinical stage, early-stage OSCC was associated with significantly better survival than advanced-stage disease, with 3-year DFS of 87.6% vs 60.3% and DSS of 93.2% vs 63.6%, respectively (Figs. 2E and 2F, p < 0.0001).

### Impact of pathological nodal status on survival

**Fig. 3:**
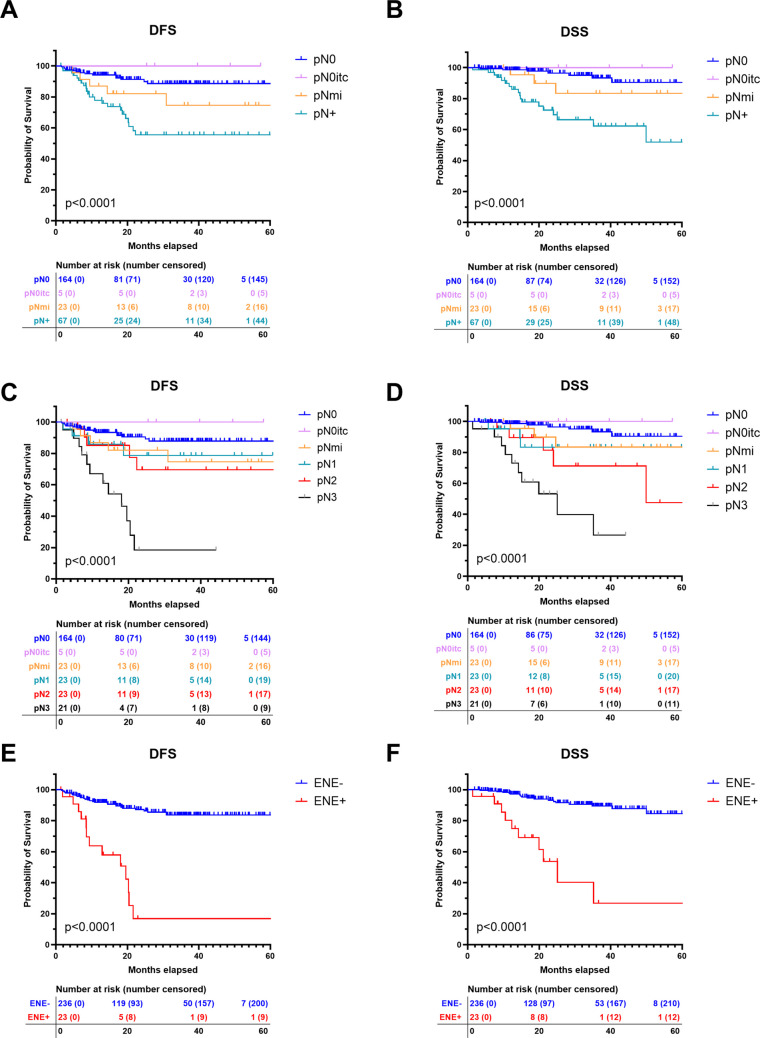
Recurrence and survival in relation to pathological lymph node status. DFS = Disease free survival. DSS = Disease specific survival. Itc = Isolated tumour cells. Mi = Micrometastasis. ENE = Extranodal extension

The presence and extent of nodal metastasis significantly influenced DFS and DSS. As shown in Fig. 3A, patients with pN0 and pN0itc status exhibited the highest probability of DFS over time, whereas those with pN + status had significantly worse survival outcomes (p < 0.0001). Patients with Mi had lower DFS and DSS than node negative cases (pN0). The 3-year DFS was 88.0% for patients with pN0, 100% for pN0itc, 73.2% for pNmi and 57.9% for patients with macrometastasis (pN +). A similar trend was observed for DSS (Fig. 3B), where patients without nodal involvement and pN0itc maintained a higher probability of 3-year DSS (93.4% and 100%, respectively) compared to those with pNmi (82.5%) and pN + (62.6%).

Further stratification of pathological nodal status (Figs. 3C and 3D) reinforced these findings. Patients with increasing nodal burden (pN1, pN2, and pN3) experienced progressively lower DFS and DSS, with pN3 patients demonstrating the worst survival outcomes (p < 0.0001).

### Regional recurrence

Out of the total 259 patients, 41 (15.8%) experienced local, regional or distant recurrence during the follow-up period. A total of 17 (6.6%) regional recurrences were observed: 8 cases (4.7%) among the 172 clinically early-stage patients and 9 cases (10.3%) among the 87 clinically advanced patients. Of the nine advanced-stage patients with regional recurrence, two were pN0, two were pN1, one was pN2, and four were pN3. Four out of the nine displayed ENE at the time of diagnosis. Among the eight early-stage cases, one was pN1 and three were pN1mi at diagnosis of the primary tumour, leaving four that were staged pN0.

Except for patients with pN2 status, recurrence rates increased with increasing nodal involvement. Regional recurrence occurred in 6/165 (3.6%) of patients with pN0 disease, 3/22 (13.6%) of patients with Mi, 3/23 (13.0%) of pN1 cases, 1/23 (4.3%) of pN2 cases and 4/21 (19.0%) of pN3 cases. Formal statistical comparison between nodal subgroups was not performed due to the limited number of events. There were no recurrences at any site in the five patients with ITCs.

### Extranodal extension and survival

ENE further stratified survival outcomes among patients with nodal involvement. As illustrated in Figs. 3E and 3 F, ENE positive (ENE +) patients had markedly lower 3-year DFS and 3-year DSS (17.2% and 26.9%, respectively) compared to ENE negative (ENE-) patients (84.6% and 89.5%, respectively) (p < 0.0001).

### Univariate and multivariate survival analysis

The univariate and multivariate analyses identified several prognostic factors influencing DFS and DSS in patients with OSCC (Tables 3 and 4). In the univariate analysis (Table 3), advanced tumour stage (pT3-4) was significantly associated with worse DFS (HR 3.337, 95% CI: 1.803–6.176, p < 0.001) and DSS (HR 5.443, 95% CI: 2.502–11.841, p < 0.001). Similarly, nodal involvement (pN +) was a strong predictor of poor prognosis, with an HR of 3.989 (95% CI: 2.054–7.774, p < 0.001) for DFS and 7.255 (95% CI: 3.0027–17.390, p < 0.001) for DSS. The presence of ENE was also associated with significantly worse outcomes (DFS: HR 7.304, 95% CI: 3.724–14.326, p < 0.001; DSS: HR 8.572, 95% CI: 3.905–18.816, p < 0.001). Additionally, patients with advanced-stage disease had a markedly increased risk of recurrence and disease-specific mortality, with HRs of 4.055 (95% CI: 2.160–7.614, p < 0.001) for DFS and 5.981 (95% CI: 2.674–13.372, p < 0.001) for DSS.

**Table 3:** Univariate analysis. DFS = Disease-free survival. DSS = Disease-specific survival. HR = Hazard ratio. 95%CI = 95% confidence interval. NE = not estimated. Itc = Isolated tumour cells. Mi = Micrometastasis. ENE = Extranodal extension. RTH = Radiotherapy. CRTH = Chemoradio-therapy. SLNB = Sentinel lymph node biopsy. ND = Neck dissection

Variable	Univariate			Univariate		
	DFS			DSS		
	HR	95%CI	p	HR	95%CI	p
**Age**						
< = 60	Ref	-	-	Ref	-	-
> 60	1,213	0,627–2,346	0,566	1,599	0,703–3,639	0,263
**Gender**						
f	Ref	-	-	Ref	-	-
m	0,907	0,522–1,779	0,907	0,990	0,683–1,437	0,959
**pT-status**						
pT1-2	Ref	-	-	Ref	-	-
pT3-4	3,337	1,803–6,176	** < 0,001**	5,443	2,502–11,841	** < 0.001**
**pN-status**						
pN0	Ref	-	-	Ref	-	-
pN0itc	NE	NE	NE	NE	NE	NE
pNmi	2,247	0,816–6,184	0,117	2,522	0,651–9,774	0,181
pN +	3, 989	2,054 −7,748	** < 0.001**	7,255	3,027–17,390	** < 0.001**
**pNx-status**						
pN0	Ref	-	-	Ref	-	-
pN1	2,216	0,970–5,066	0,057	2,888	0,969–8,609	0,057
pN2	2,682	0,974–7,384	0,056	5,881	1,862–18,574	**0,003**
pN3	9,822	4,540–21,249	** < 0,001**	17,383	6,536–46,235	** < 0.001**
**ENE**						
ENE-	Ref	-	-	Ref	-	-
ENE +	7,304	3,724–14,326	** < 0,001**	8,572	3,905–18,816	** < 0.001**
**Stage**						
Early	Ref	-	-	Ref	-	-
Advanced	4,055	2,160–7,614	** < 0,001**	5,981	2,674–13,379	** < 0.001**
**Surgical treatment**						
SLNB only	Ref	-		Ref		
ND + SLNB	2,566	1,285–5,121	**0,008**	3,794	1,438–10,012	**0,007**
**Adjuvant treatment**						
Surgery only	Ref			Ref		
Surgery + RTH	1,821	0,852–3,888	0,122	2,770	0.986–7,778	0,053
Surgery + CRTH	6,756	3,017–15,130	** < 0,001**	10,848	3,695–31,852	** < 0,001**

**Table 4:** Multivariate analysis. DFS = Disease-free survival. DSS = Disease-specific survival. HR = Hazard ratio. 95%CI = 95% confidence interval. NE = not estimated. Itc = Isolated tumour cells. Mi = Micrometastasis. RTH = Radiotherapy. CRTH = Chemoradiotherapy

Variable	Multivariate			Multivariate		
	DFS			DSS		
	HR	95%CI	p	HR	95%CI	p
**pT-status**						
pT1-2	Ref	-	-	Ref	-	-
pT3-4	2,188	1,101–4,346	**0,025**	3,191	1,361–7,481	**0,008**
**pN-status**						
pN0	Ref	-	-	Ref	-	-
pN0itc	NE	NE	NE	NE	NE	NE
pNmi	2,351	0,843–6,557	0,102	2,572	0,657–10,066	0,175
pN +	2,341	1,032 −5,311	**0,042**	4,320	1,590–11,733	**0,004**
**Adjuvant treatment**						
Surgery only	Ref			Ref		
Surgery + RTH	0,995	0,427–2,321	0,991	1,018	0,328–3,157	0,975
Surgery + CRTH	2,302	0,796–6,657	0,124	1,917	0,522–7,034	0,327

Multivariate analysis confirmed that pT3-4 status (DFS: HR 2.188, 95% CI: 1.101–4.346, p = 0.025; DSS: HR 3.191, 95% CI: 1.361–7.481, p = 0.008) and pN + status (DFS: HR 2.341, 95% CI: 1.032–5.311, p = 0.042; DSS: HR 4.320, 95% CI: 1.590–11.733, p = 0.004) remained independent predictors of poor outcomes. While pNmi showed a trend toward worse survival, it did not reach statistical significance in either DFS (HR 2.351, 95% CI: 0.843–6.557, p = 0.102) or DSS (HR 2.572, 95% CI: 0.657–10.066, p = 0.175).

## Discussion

Lymph node metastasis is common in head and neck cancer and has long been recognized as a major risk factor for recurrence and mortality [9, 29]. The findings of the current study are consistent with previous reports demonstrating the prognostic significance of both clinical and pathological tumour stage, nodal involvement and ENE in OSCC. Furthermore, a trend toward reduced DFS and DSS was observed in patients with Mi, whose survival and recurrence outcomes were comparable to those with N1 disease. In contrast, ITCs did not appear to impact survival or recurrence. Although regional recurrence was rare, it was more common in clinically advanced-stage disease, and increased with increasing nodal involvement. Our results demonstrate that increasing nodal burden is strongly associated with poorer DFS and DSS, reinforcing the well-established role of nodal status as a marker of adverse prognosis in OSCC [30].

The use of SLNB has become well established for early-stage OSCC [12]. At Karolinska University Hospital it is also included in the management protocol for patients with advanced-stage disease. Some institutions perform imaging within 5 to 30 min after radiotracer injection. This method aims to identify the first lymph nodes the tumour drains to – the sentinel nodes (SNs) – while potentially reducing the number of negative nodes resected [16, 31]. Others opt for longer intervals to detect additional nodes and obtain more precise anatomical information [32, 33] as there is evidence that suggests that SNs with weaker signals may contain metastases [34]. At our institution, SPECT-CT is performed no earlier than one hour after peritumoral injection of technetium-99 (Tc-99m)-labelled tracer [28]. The extended imaging interval is intended to identify all tumour draining lymph nodes (TDLNs), not just first-echelon SNs. This approach may reduce the rate of metastasis to non-sentinel nodes [12].

The clinical relevance of Mi and ITCs in OSCC remains controversial, with no universally accepted guidelines for the management of these microscopic tumour deposits. In the present study, 20.1% of patients were upstaged from cN0 to either pN + or pNmi following SLNB. Among those with early-stage disease, 11.0% were found to have only Mi, compared to 3.4% of cases with advanced-stage disease. Patients with Mi demonstrated DFS and DSS rates similar to those with pN1 status, whereas ITC did not appear to impact either outcome. These findings support classifying Mi as metastatic disease, and managing it accordingly in the post-operative setting. They also reinforce the value of SLNB across all stages of OSCC, particularly in node-negative disease.

Although the observed trends suggest potentially meaningful differences, statistical significance was not reached. This is likely in part due to limited statistical power, resulting from the small size of the Mi and ITC subgroups (n = 22 and 5, respectively). The biological basis for the apparent difference between the two groups is not yet fully understood. It is, however, plausible that the immune system more effectively eliminates ITCs because of the lower number of tumour cells involved. Mi, on the other hand, have not only spread to the lymph node, but also formed a larger metastatic deposit, suggesting greater malignant potential. Moreover, as the definitions of Mi and ITC were originally established for breast cancer [18], the cut-off sizes for Mi and ITC may not be optimal when applied to OSCC. While not statistically analysed further in this study, treatment-related differences do not appear to explain the discrepancy. Four of five patients (80.0%) with ITCs did not receive adjuvant treatment, yet there were no cases of disease recurrence or death.

These findings align with some previous reports suggesting reduced survival in OSCC patients with nodal micrometastases. A recent systematic review including 16 studies investigated the clinical significance of Mi and ITC in oral and oropharyngeal SCC (OOSCC), yielding heterogeneous results [4]. Some studies conducted more extensive analyses of Mi/ITC separately using univariate and/or multivariate models. Three prospective studies [5, 27, 35] and one retrospective study [26] demonstrated a significant impact of Mi on DSS or overall survival (OS). Conversely, one retrospective study [25] found no association with either OS or DFS. As in previous studies, the current sample size is limited. In addition, differences in other disease-related characteristics or unmeasured clinicopathological factors that influence prognosis cannot be excluded. Nevertheless, the observed trend toward poorer survival in patients with Mi may justify the consideration of adjuvant therapy, pending confirmation in larger, prospective studies.

Evidence regarding ITCs is even more limited. Multiple studies, including the present one, have not demonstrated a significant effect of ITCs on survival or recurrence [5, 6, 24, 26]. However, one study reported that among five patients with only ITCs in their sentinel lymph node biopsy (SLNB), two experienced disease recurrence despite subsequent neck treatment, leading the authors to conclude that ITCs should not be disregarded [34]. Our results do not support this conclusion, as the five patients with nodal ITCs exhibited a 100% survival rate and no recurrences during the follow-up period. Thus, ITCs may not need to be treated as metastatic disease. Nevertheless, there were only five patients in the ITC subgroup, limiting the statistical power of the survival analysis. Therefore, these findings should be interpreted with caution. Further studies are required to determine whether ITCs can be safely disregarded or should instead be managed as metastatic disease.

The presence of ENE was associated with significantly poorer rates of survival and recurrence. This finding is consistent with existing literature [8–11], which highlights ENE as a critical marker for identifying high-risk patients, with some reports suggesting it may reduce survival rates by 50% [11]. In the present study, DFS and DSS were reduced by as much as 80% and 70%, respectively, compared to ENE- patients. ENE has even been added as a prognostic variable incorporated into the TNM classification of head and neck tumours [20, 36]. Alongside pN3 status, ENE was among the strongest predictors of adverse outcomes in the current study. The poor prognosis observed in these high-risk patients may support the need for more aggressive adjuvant therapy, including the potential use of adjuvant immunotherapy.

### Limitations

This study has several limitations. First, the sample size is small overall, particularly within the Mi (n = 22) and ITC (n = 5) subgroups. The limited number of cases reduces statistical power to detect survival differences and contributes to wide confidence intervals; therefore, subgroup findings should be interpreted with caution. In addition, the number of variables included in the multivariate analyses was limited, which may have affected the interpretation of tumour stage, nodal burden, and ENE as independent prognostic risk factors. Second, the single-centre, retrospective design may introduce selection bias and limits external generalizability. Although patients with less than two years of follow-up were censored at their last recorded visit in the time-to-event analyses, shorter follow-up may have limited the detection of recurrences occurring beyond the available follow-up period. Third, there is potential heterogeneity in adjuvant treatment (e.g., indications, timing, and modality), which could influence outcomes and complicate comparisons between groups. Finally, despite adjustment for available covariates, unmeasured or incompletely measured confounding (including missing data on factors such as smoking) may persist. Furthermore, some potentially important clinicopathological variables and prognostic factors, such as the Charlson Comorbidity Index (CCI) score, nodal yield from neck dissection, surgical margins, and depth of invasion were not available at the time of the study. Future research should prioritize larger, prospective, ideally multicenter cohorts and more standardized adjuvant treatment protocols to validate these observations and refine the management of Mi and ITC in OSCC.

## Conclusions

Clinical and pathological tumour stage, extent of nodal involvement and presence of ENE are key prognostic factors for survival and recurrence in OSCC. Both ENE + and pN3 status were associated with high rates of mortality and recurrence. DFS and DSS rates in patients with pNmi were comparable to those observed in pN1 disease, whereas ITCs had no impact on either outcome. These findings support the classification of Mi as metastatic disease and underscore the importance of SLNB in the management of early- and advanced-stage OSCC.
